# Associations of exercise procrastination and exercise addiction with mental well-being

**DOI:** 10.3389/fpsyg.2026.1818526

**Published:** 2026-05-08

**Authors:** Wenbo Ma, Yanxia Zhao, Hengzhi Deng, Yike Li, Zhengyang Mei, Lunxin Chen, Chao Wang, Xing Zhang, Weihua Zheng, Hansen Li

**Affiliations:** 1 School of Physical Education, Henan University of Economics and Law, Zhengzhou, China; 2 College of Physical Education, Chongqing University, Chongqing, China; 3 Faculty of Sports and Exercise Science, University of Malaya, Kuala Lumpur, Malaysia; 4 College of Physical Education, Southwest University, Chongqing, China; 5 School of Physical Education and Sports, Central China Normal University, Wuhan, China; 6 Department of Physical Education, Xinjiang Hetian College, Hetian, China; 7 Department of Physical Education and Sport, Faculty of Sport Sciences, University of Granada, Granada, Spain; 8 School of Social Sports, Shenyang Sport University, Shenyang, China; 9 School of Physical Education, Sichuan Agricultural University, Yaan, China

**Keywords:** exercise addiction, exercise procrastination, mental well-being, physical exercise, university students

## Abstract

Exercise procrastination and exercise addiction reflect maladaptive patterns that may be linked to mental well-being, yet their dose–response relationships and potential mechanisms remain unclear. We conducted a cross-sectional survey among 570 Chinese university students (mean age = 19.15 ± 1.09 years; 52.3% men). Exercise procrastination was assessed using the Procrastination in Exercise Scale (PES), exercise addiction using the Revised Exercise Addiction Inventory (EAI-R), physical activity using the Physical Activity Rating Scale (PARS-3), and mental well-being using the WHO-5 Well-Being Index. Pearson correlations were used to describe bivariate associations. Restricted cubic spline (RCS) models were applied to examine potential non-linear associations between exercise procrastination/addiction and mental well-being. Mediation analyses were conducted as a theory-informed secondary analysis to examine whether physical activity might represent a potential pathway linking these associations. We found that exercise addiction was negatively correlated with mental well-being, while exercise procrastination was positively correlated with mental well-being. RCS analyses indicated a significant association between exercise addiction and mental well-being with weak evidence of non-linearity. For exercise procrastination, the association with mental well-being showed an inverted U-shaped pattern. In mediation models, physical activity did not significantly mediate the associations between exercise addiction/procrastination and mental well-being. In conclusion, exercise addiction and exercise procrastination show distinct patterns of association with mental well-being, and these relationships were not explained by physical activity in this sample. Future research should further clarify alternative psychological or contextual pathways linking these exercise-related behaviors to mental well-being.

## 1 Introduction

Physical activity is a cornerstone of physical health and an important lifestyle determinant of mental well-being (Fox, 1999). However, global levels of physical activity remain suboptimal (Strain et al., 2024; Guthold et al., 2018). In this context, increasing population-level physical activity is a key public-health objective. At the same time, physical activity is not invariably “the more, the better.” For sedentary people, increasing activity is clearly beneficial; however, among highly active individuals, excessive training may contribute to performance decrements, injury, and endocrine-related problems (Brenner and Watson, 2024; Gleeson, 2002).

Between the extremes of inactivity and excessive exercise, two maladaptive exercise-related patterns may be relevant to mental well-being: exercise procrastination and exercise addiction. Exercise procrastination refers to the repeated delay of intended exercise despite having already formed an exercise plan and in the absence of clear external barriers, such as injury or unavoidable time constraints (Kelly and Walton, 2021).

More specifically, exercise procrastination may be conceptualized as a domain-specific form of health behavior procrastination. Its defining feature is not merely doing little exercise, but voluntarily delaying the enactment of intended exercise despite recognizing its value and despite the absence of major external constraints. This distinction is important because a person may be insufficiently active without procrastinating on exercise at all, for example because they never formed an intention to exercise, face genuine environmental or physical barriers, or deliberately reschedule exercise for reasonable reasons. By contrast, exercise procrastination refers to a specific intention–behavior failure within the exercise domain. This view is also consistent with broader work suggesting that procrastination can vary across life domains and with health behavior procrastination research emphasizing that people may knowingly postpone intended health-promoting actions even when they endorse their long-term benefits (Klingsieck, 2013; Kroese and de Ridder, 2016). In support of its distinctiveness, Kelly and Walton (2021) found that exercise procrastination predicted lower physical activity even after controlling for exercise intentions and general procrastination, suggesting that it is not reducible to either low activity or a general procrastinating tendency.

Notably, exercise procrastination may not be necessarily negative. Affect-regulation accounts propose that procrastination often serves as a short-term mood repair strategy, whereby individuals delay an intended action to reduce immediate negative affect, such as dread, stress, fatigue, guilt, or anticipated discomfort (Sirois and Pychyl, 2013; Pychyl and Sirois, 2016). In the exercise domain, postponing a planned workout may therefore provide temporary emotional relief, especially when exercise is construed as effortful, obligatory, or aversive. Accordingly, very low levels of exercise procrastination may not always be uniformly maladaptive and may, in some situations, reflect temporary emotional coping or flexible adjustment.

On the other hand, exercise addiction is usually conceptualized as a compulsive and psychologically embedded pattern of exercise involvement, characterized by loss of control, conflict, rigid persistence, and continued engagement despite negative consequences (Lichtenstein et al., 2017).

Recent work further suggests that exercise addiction may be rooted in broader psychological vulnerabilities rather than being solely a behavioral phenomenon. For example, Arayici and Sutcu (2024) conceptualized exercise addiction within a broader psychological framework and showed that basic psychological needs and emotion regulation difficulties were closely associated with exercise addiction symptoms.

This perspective is broadly consistent with and partly informed by Self-Determination Theory (SDT), which identifies autonomy, competence, and relatedness as three universal basic psychological needs and conceptualizes them as key sources of human motivation (Deci and Ryan, 2013; Ryan and Deci, 2008; Ryan and Deci, 2000). Applied to exercise, SDT suggests that the maintenance of a healthy relationship with physical activity depends not only on whether individuals exercise, but also on whether exercise involvement is supported by need satisfaction and self-determined motivation. When people experience lower autonomy, reduced competence in managing their exercise behavior, or weaker relatedness in exercise settings, exercise may be more likely to take on a compensatory or controlled function. Under such conditions, individuals may become increasingly reliant on exercise as a way of regulating distress or compensating for unmet psychological needs, rather than engaging in exercise in a flexible and self-endorsed manner. In this sense, exercise addiction may reflect not merely excessive enthusiasm for exercise, but a dysregulated form of self-regulation rooted in motivational imbalance and psychological vulnerability (González-Cutre and Sicilia, 2012; Edmunds et al., 2006; Arayici and Sutcu, 2024).

Relatedly, the dualistic model of passion helps distinguish healthy from maladaptive exercise involvement. According to this perspective, harmonious passion reflects an autonomous internalization of an activity, such that the individual remains in control and the activity stays compatible with other life domains. Obsessive passion, in contrast, reflects a controlled internalization that creates internal pressure to engage, conflict with other valued domains, and rigid persistence even when the activity becomes costly (Vallerand et al., 2003). Applied to exercise, this distinction suggests that not all frequent or highly valued exercise should be regarded as pathological. The key issue is whether exercise remains flexible, self-endorsed, and integrated into life, or becomes compulsive, inflexible, and psychologically harmful.

This distinction is important when considering its association with mental well-being. Individuals who are highly engaged in exercise for autonomous and flexible reasons may maintain or even enhance well-being, whereas individuals whose exercise is driven by compulsive persistence or affect-regulatory needs may be more vulnerable to poorer mental well-being.

Accordingly, we investigated the associations of exercise procrastination and exercise addiction with mental well-being and preliminarily examined the mediating role of physical activity among Chinese university students. We hypothesized that exercise procrastination would show a potential non-linear association with mental well-being, whereas exercise addiction would be negatively associated with mental well-being. We further examined whether physical activity might partially explain these associations. We chose university students as the study population for two main reasons. First, this group was feasible and convenient to access for recruitment. Second, compared with other educational stages, Chinese students at the university level generally have the greatest amount of discretionary time, which may allow a wider range of behavioral patterns to emerge. Engaging in exercise is one possibility, whereas sedentary behaviors related to emerging digital media use, such as short-video viewing and video gaming, represent another. As a result, behavioral polarization may be more pronounced in this population than in other groups.

We hypothesized that: (1) exercise addiction and exercise procrastination would show negative associations with mental well-being only beyond certain ranges (i.e., non-linear associations); and (2) physical activity would partially explain these associations.

## 2 Methods

### 2.1 Participants

University students were recruited in public campus areas over 2 weeks starting on 1 November 2024. Research staff approached potential participants and guided them to complete an online questionnaire on their smartphones. Inclusion criteria were: (a) registered university student; and (b) sufficient physical capability to participate in standard physical education courses. Exclusion criteria were: (a) abnormally fast completion time; (b) incomplete responses; and (c) extreme values in open-ended numeric items (screened using the interquartile range method). Participants provided written informed consent before participation. A total of 570 students completed the survey. The study was approved by the Ethics Committee of College of Physical Education at Southwest University.

Although the present study did not use a probability-based sampling design, we still used a conventional prevalence-based formula to obtain a rough reference for the minimum sample size required. The formula, which is commonly used in cross-sectional studies for proportion estimation, is as follows (Pourhoseingholi et al., 2013; Tumuhamye et al., 2013; Charan and Biswas, 2013):


$$N=\frac{p(1-p)Z^{2}}{d^{2}}$$ (1)

In the formula, *N* denotes the required sample size, while *p* represents the prevalence of the target variable. Z corresponds to the z-score associated with the desired confidence level, and *d* indicates the level of precision (i.e., the acceptable margin of error or effect size). Most researchers report their results using a 95% confidence interval (CI), in which case *Z* is typically set to 1.96. When the prevalence of the condition ranges between 10 and 90%, some scholars recommend setting the precision (*d*) at 5% (Pourhoseingholi et al., 2013).

There are currently no published estimates of the prevalence of exercise procrastination. Therefore, we based our calculation on the reported prevalence of exercise addiction (approximately 5.5%; Trott et al. (2020)), which yielded a minimum required sample size of 80 participants.

### 2.2 Variables and measurements

#### 2.2.1 Exercise procrastination (EP)

Exercise procrastination was measured using the Procrastination in Exercise Scale (PES) (Kelly and Walton, 2021). The PES includes six items loading on a single factor. Items are rated on a five-point Likert scale (1 = strongly disagree, 5 = strongly agree), with higher scores indicating greater procrastination. Following cross-cultural translation guidance (Cheung et al., 2020), we translated the PES into Chinese. The pre-validation of this instrument in our target population can be found in our previous publication (Liao et al., 2025). Internal consistency in this study was high (Cronbach’s *α* = 0.90). The sum of scores across all items was used to represent the overall exercise procrastination level.

#### 2.2.2 Exercise addiction (EA)

Exercise addiction was assessed using the Revised Exercise Addiction Inventory (EAI-R), an improved version of the original EAI (Szabo et al., 2019). The EAI-R contains six items rated on a six-point Likert scale (1 = completely disagree, 6 = completely agree), with higher scores indicating stronger addiction tendency. The validated Chinese version we used retains five items and is unidimensional among Chinese college students (Wang Y. et al., 2024). Internal consistency was acceptable (Cronbach’s *α* = 0.80). The sum of scores across all items was used to represent the overall exercise addiction level.

#### 2.2.3 Physical activity (PA)

Physical activity was measured with the Physical Activity Rating Scale-3 (PARS-3). The instrument was developed by Hashimoto (2005) and adapted for Chinese populations by Liang (1994). PARS-3 assesses intensity, duration, and frequency, each with one item rated on a five-point Likert scale. Higher scores reflect greater physical activity. The total score was computed using the standard PARS-3 formula: Multiply scores from each component (Intensity × (Time − 1) × Frequency). Intensity/Frequency (1–5 points), Time (0–4 points).

#### 2.2.4 Mental well-being

Mental well-being was assessed using the WHO-5 Well-Being Index, a brief self-report measure of subjective well-being over the past 2 weeks (Topp et al., 2015). It consists of five positively worded items (e.g., feeling cheerful, calm/relaxed, active/vigorous, waking up refreshed, and being interested in daily life). Each item is rated from 0 to 5 (from “at no time” to “all of the time”). Item scores are summed to yield a raw score from 0 to 25; the raw score can be multiplied by four to obtain a 0–100 scale, with higher values indicating better well-being. The WHO-5 has been previously translated into Chinese (Fung et al., 2022) and has been widely used in Chinese samples (Jiang et al., 2025; Li et al., 2024a, 2024b; Li et al., 2023), and internal consistency in this study was acceptable (Cronbach’s *α* = 0.80).

#### 2.2.5 Covariates

We adjusted analyses for sex, age, grade level, major, and monthly household income. Age was recorded as an integer (years). Sex was coded male = 0 and female = 1. Grade level was coded 1 = first year, 2 = second year, 3 = third year, 4 = fourth year, 5 = postgraduate. Major was coded 0 = natural sciences and 1 = humanities and social sciences. Monthly household income was coded as: 1 = 0–5,000 RMB; 2 = 5,001–10,000 RMB; 3 = 10,001–15,000 RMB; 4 = 15,001–20,000 RMB; 5 = 20,001–25,000 RMB; 6 = 25,001–30,000 RMB; 7 ≥ 30,000 RMB.

### 2.3 Statistical analysis

#### 2.3.1 Correlation analysis

Pearson correlations were used to describe bivariate associations among EA, EP, PA, and mental well-being. To illustrate score differences among students, we used independent-samples Kruskal–Wallis tests and independent-samples Mann–Whitney *U* tests to compare scores across sex, major, and grade groups.

#### 2.3.2 Nonlinear trend assessment

To evaluate potential dose–response relationships of EA, EP, and PA with mental well-being, we used restricted cubic spline (RCS) regression in fully adjusted models (Desquilbet and Mariotti, 2010). Four knots were specified at the 5th, 35th, 65th, and 95th percentiles. This number of knots balances sensitivity to the underlying trend with protection against overfitting and is a common choice in many studies (Xu et al., 2025; Zhang et al., 2025). Wald tests assessed both the overall association and departures from linearity (Xiaolan et al., 2024). Covariates were included as described above; binary covariates were entered directly, and grade level and income were treated as categorical factors in the RCS models. Analyses were conducted using the RMS package in R (v4.3.1).

#### 2.3.3 Mediation analysis

As a theory-informed secondary analysis, we examined whether physical activity might function as a potential mediator of the associations of EA and EP with mental well-being. Given that the physical activity–mental well-being association may vary across contexts and domains, these mediation analyses were intended as exploratory tests of a plausible mechanism rather than definitive evidence of explanation. When nonlinearity was indicated, we conducted segmented mediation analyses based on the RCS-derived turning point, following prior work (Wang F. et al., 2024; Li et al., 2025). Models were estimated with maximum likelihood, and bias-corrected bootstrapping (Hayes and Scharkow, 2013) with 10,000 resamples was used to obtain confidence intervals (Haukoos and Lewis, 2005; Kelley, 2005; Brown, 2015). Because the simple mediation models were saturated, model fit indices are not reported; interpretation focuses on path estimates. For mediation, an indirect effect (product of constituent paths) significantly different from zero was considered evidence of mediation (Hayes, 2017; Zhao et al., 2010). In mediation models, grade level and income were entered as continuous covariates to satisfy modeling constraints and to estimate average effects per unit increase. The mediation analyses were performed in AMOS 26. A two-tailed *p*-value <0.05 was considered statistically significant.

## 3 Results

### 3.1 Participant characteristics

The analytic sample included 570 students (mean age = 19.15 ± 1.09 years; median = 19, IQR = 2). Men comprised 52.3% (*n* = 298) and women 47.7% (*n* = 272). Most participants were in Year 1 (58.2%), followed by Year 2 (22.8%), Year 3 (15.3%), and Year 4 (3.7%). Majors were evenly distributed between natural sciences (49.1%) and humanities/social sciences (50.9%). The largest income categories were 0–5,000 RMB (41.9%) and 5,001–10,000 RMB (33.3%). Mean (SD) values were 16.74 (5.83) for exercise addiction, 16.98 (5.99) for exercise procrastination, 23.09 (22.87) for physical activity, and 13.46 (4.90) for mental well-being (see Table 1).

**Table 1:** Participant characteristics.

Variable	Category/unit	*n* (%)	Mean (SD)	Median (IQR)
Sex	Male	298 (52.3)	—	—
	Female	272 (47.7)	—	—
Grade level	Year 1	332 (58.2)	—	—
	Year 2	130 (22.8)	—	—
	Year 3	87 (15.3)	—	—
	Year 4	21 (3.7)	—	—
Major	Natural sciences	280 (49.1)	—	—
	Humanities and social sciences	290 (50.9)	—	—
Income (RMB)	0–5,000	239 (41.9)	—	—
	5,001–10,000	190 (33.3)	—	—
	10,001–15,000	73 (12.8)	—	—
	15,001–20,000	35 (6.1)	—	—
	20,001–25,000	18 (3.2)	—	—
	25,001–30,000	1 (0.2)	—	—
	>30,000	14 (2.5)	—	—
Age (years)	—	—	19.15 (1.09)	19 (2)
Exercise addiction	—	—	16.74 (5.83)	16 (7)
Exercise procrastination	—	—	16.98 (5.99)	18 (6)
Physical activity	—	—	23.09 (22.87)	16 (32)
Mental well-being	—	—	13.46 (4.90)	12 (6)

Scores varied modestly across demographic groups. Social science students showed higher EAI and PA scores than natural science students, while no major-based differences were observed for EP or WHO-5. By sex, male students had higher EAI and PA scores, whereas female students had higher EP scores; WHO-5 did not differ significantly between men and women. Across grades, EAI and WHO-5 scores were broadly similar, but EP and PA showed significant variation, with Year 2 and Year 3 students reporting higher EP and PA than Year 1 students (Supplementary Tables S1–S3).

### 3.2 Correlations among study variables

Pearson correlations are shown in Table 2. Exercise addiction was positively correlated with exercise procrastination (*r* = 0.230, *p* < 0.001) and physical activity (*r* = 0.269, *p* < 0.001), and negatively correlated with mental well-being (*r* = −0.162, *p* < 0.001). Exercise procrastination was negatively correlated with physical activity (*r* = −0.191, *p* < 0.001) and weakly positively correlated with mental well-being (*r* = 0.127, *p* = 0.002). Physical activity was not significantly correlated with mental well-being (*r* = −0.041, *p* = 0.331).

**Table 2:** Pearson correlations.

Variable	Statistic	EA	EP	PA	Mental well-being
EA	*r*	1	—	—	—
	*p*	—	—	—	—
EP	*r*	0.230	1	—	—
	*p*	<0.001	—	—	—
PA	*r*	0.269	−0.191	1	—
	*p*	<0.001	<0.001	—	—
Mental well-being	*r*	−0.162	0.127	−0.041	1
	*p*	<0.001	0.002	0.331	—

EA, exercise addiction; EP, exercise procrastination; PA, physical activity.

### 3.3 Nonlinear associations

**Figure 1:**
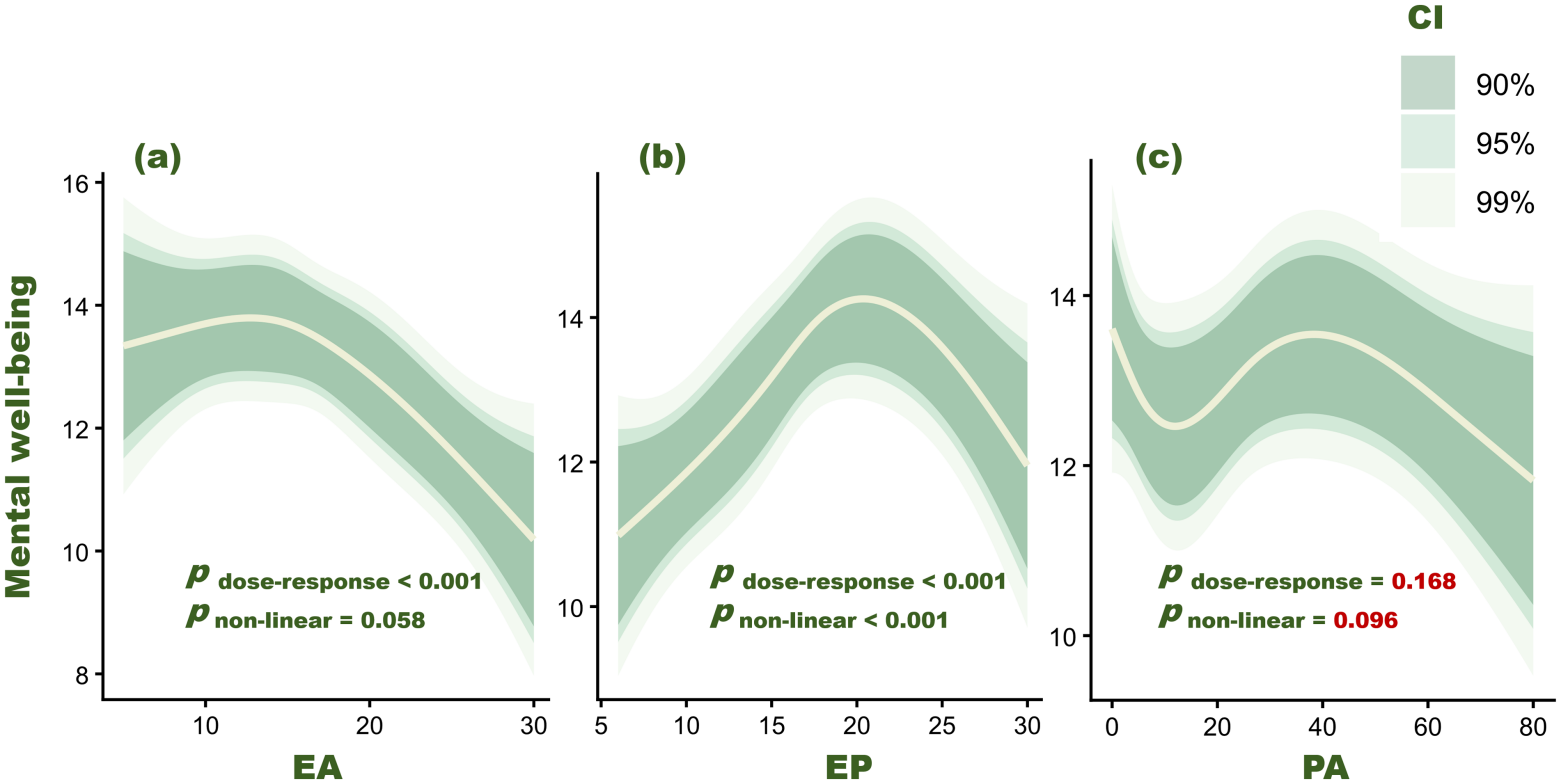
Associations of exercise addiction **(a)**, exercise procrastination **(b)**, and physical activity **(c)** with mental well-being. *p*-values correspond to Wald *χ*^2^ tests for overall associations and departures from linearity.

RCS models indicated a significant overall association between exercise addiction and mental well-being (*p* < 0.001), with only weak evidence of nonlinearity (*p* = 0.058). In contrast, exercise procrastination was significantly associated with mental well-being (*p* < 0.001) and showed clear nonlinearity (*p* < 0.001). The predicted curve suggested a turning point at EP = 20.351, consistent with an inverted U-shape (mental well-being increased at lower EP levels but decreased at higher levels). This turning point was used to stratify EP in subsequent segmented models (see Figure 1).

### 3.4 Mediation analysis

**Figure 2:**
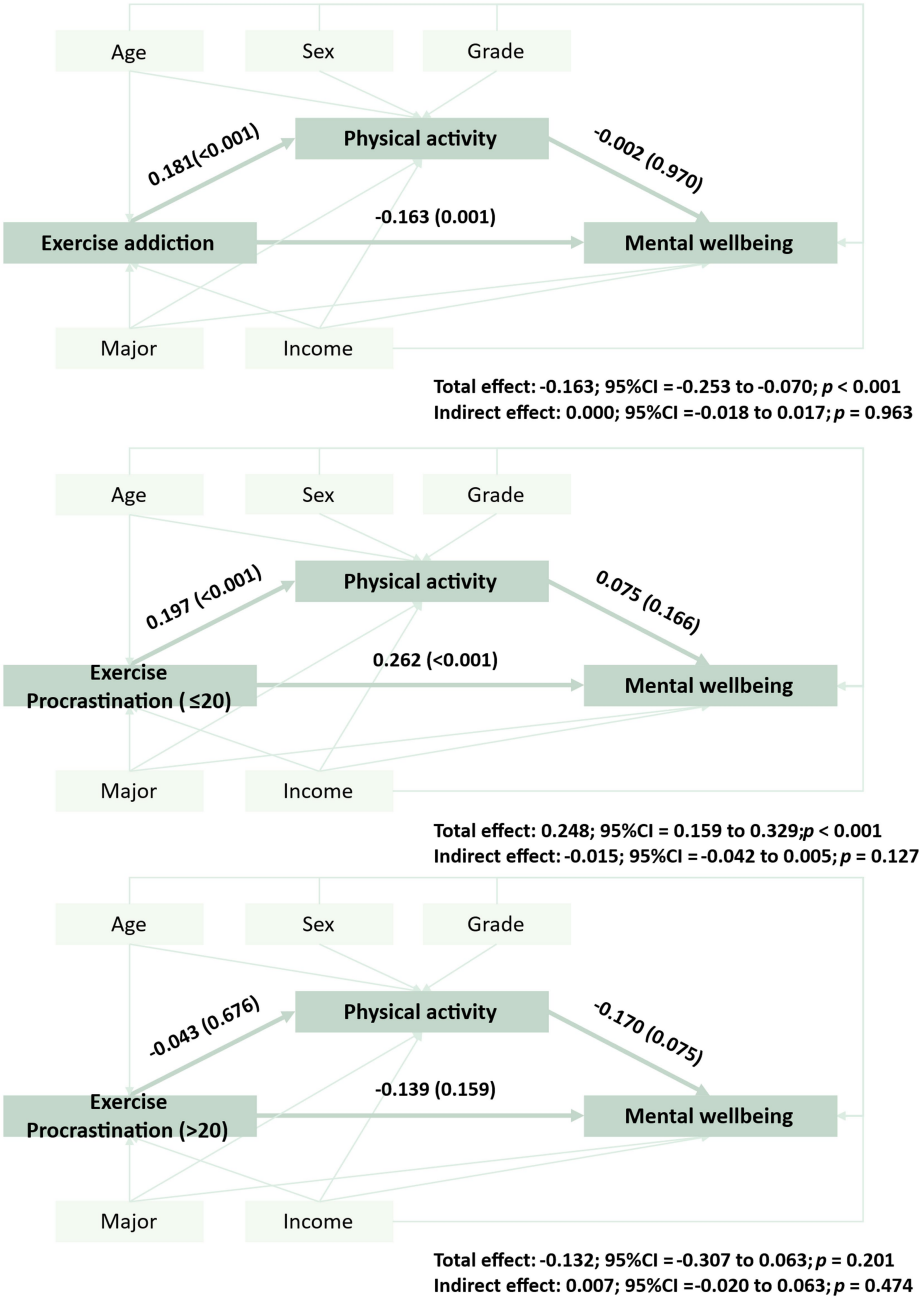
Mediation model linking exercise addiction and exercise procrastination to mental well-being via physical activity. **(a)** shows the model with exercise addiction as the independent variable, whereas **(b,c)** show models with exercise procrastination of different score ranges as the dependent variable. Red lines indicate non-significant pathways between variables. The numbers outside the parentheses represent standardized regression coefficients, whereas those inside the parentheses represent *p*-values.

For exercise addiction, the total association with mental well-being was significant (*β* = −0.163, *p* < 0.001). However, the indirect effect via physical activity was not significant because the PA → mental well-being path was not significant, although the EA → PA path was significant. For exercise procrastination, segmented mediation analyses were performed. In the EP ≤ 20 subgroup (*N* = 434), EP showed a positive association with mental well-being only at a statistically significant threshold (*p* < 0.001). In the EP > 20 subgroup (*N* = 136), the EP → mental well-being association was negative but not significant (*p* = 0.201). In both subgroups, there was no evidence of mediation via physical activity (see Figure 2).

## 4 Discussion

### 4.1 Summary of findings

This study examined exercise addiction and exercise procrastination in relation to mental well-being among university students and explored whether physical activity explained these associations. RCS analyses suggested an approximately linear negative association between exercise addiction and mental well-being and an inverted U-shaped association between exercise procrastination and mental well-being. No mediation via physical activity was detected for either construct, which did not support our second hypothesis.

### 4.2 Comparison with prior research

We observed an approximately linear negative association between exercise addiction and mental well-being. Previous surveys conducted in high-volume exercise populations have shown a significant positive association between the number of mental disorders—including depression, personality disorders, and obsessive-compulsive disorder—and the severity of exercise addiction (Meyer et al., 2021). Systematic reviews have likewise indicated that individuals at risk of exercise addiction are more likely to exhibit symptoms of mental disorders than those without such risk (Colledge et al., 2020). In contrast to these studies, our focus was not on the number or probability of psychiatric diagnoses, but on the association between mental health scores and exercise addiction scores. We initially hypothesized that mild exercise addiction might not necessarily be associated with poorer mental health and could, in some contexts, coincide with sustained physical activity despite certain activity-related barriers (e.g., mild physical discomfort or chronic symptoms that do not pose a substantial threat). However, we observed only an approximately linear negative association between exercise addiction and mental health. In other words, this relationship appears to be nearly monotonic, suggesting that exercise addiction is detrimental—rather than beneficial—to mental health across its entire range.

A point that should be considered is that exercise addiction may reflect not only maladaptive exercise involvement per se, but also broader psychological vulnerability. Prior literature has linked exercise addiction with eating-disorder pathology, obsessive-compulsive symptoms, depression, anxiety, emotional dysregulation, and clinically relevant psychiatric burden (Lichtenstein et al., 2017; Meyer et al., 2025; Wang et al., 2026). Emerging evidence also suggests that childhood trauma and problematic family experiences may be associated with exercise addiction risk, partly through emotional dysregulation and body-image-related pathways (Sweetnam and Flack, 2023; Zhang and Xue, 2026; Colledge et al., 2021). Because the present study did not assess trauma history, emotional dysregulation, or prior psychopathology, these factors could not be examined directly here. They may nevertheless represent important background variables that help explain why some individuals show more compulsive and potentially harmful exercise patterns than others.

On the other hand, our correlation analyses showed a weak positive association between exercise procrastination and mental well-being, whereas the RCS models suggested an inverted U-shaped relationship. Taken together, these findings indicate that the association may not be adequately captured by a simple linear summary. Importantly, this pattern should not be interpreted as suggesting that exercise procrastination is beneficial in itself. A more cautious interpretation is that very low levels of exercise procrastination may, in some situations, reflect temporary emotional coping, flexible postponement, or the absence of persistent self-regulatory conflict, whereas higher levels may reflect a more stable intention–behavior failure that interferes with exercise enactment and is more likely to accumulate into guilt, self-blame, and goal conflict over time. From this perspective, the left side of the curve may reflect relatively non-pathological delay, whereas the right side may reflect more persistent and maladaptive procrastination.

In the exercise context, postponing a planned workout may therefore be temporarily relieving, especially when exercise is perceived as effortful, obligatory, or aversive (Pychyl and Sirois, 2016). However, when such delay becomes repeated and habitual, its short-term affective benefits may be outweighed by longer-term costs, including reduced exercise enactment, self-regulatory conflict, and poorer mental well-being. Thus, the apparent inverted U-shape may reflect a shift from occasional or flexible delay to more persistent and maladaptive procrastination.

At the same time, this explanation should be interpreted cautiously. The segmented analyses did not yield statistically significant linear associations on both sides of the turning point at the conventional *p* < 0.05 threshold, although the regression coefficients were directionally consistent with the spline pattern. Therefore, the segmented analyses should be viewed as exploratory follow-up analyses rather than as independent confirmation of the inverted U-shaped relationship. In addition, because the present study was cross-sectional, reverse causation cannot be ruled out. For example, students with better mental well-being may be more tolerant of occasional exercise delay and less likely to experience such delay as distressing, whereas students with poorer well-being may be more vulnerable to repeated self-regulatory failure. Accordingly, the inverted U-shaped association should be regarded as a preliminary pattern requiring replication in longitudinal or intensive repeated-measures research.

It should be also noted that the discrepancy in statistical significance between these two analytical approaches may be attributable to differences in sample size, as the RCS analyses were based on the full sample (*N* = 570), whereas the segmented mediation regressions relied on two smaller subsamples (*N* = 434 and *N* = 136). The reduced sample sizes likely diminished statistical power.

Finally, contrary to our initial hypothesis, we did not observe evidence that physical activity mediated the associations of exercise addiction or exercise procrastination with mental well-being. Although exercise addiction and exercise procrastination were significantly associated with physical activity in opposite directions, physical activity was not significantly associated with mental well-being in our data, which limits its interpretability as a mediator in this sample. Therefore, physical activity should not be considered a supported explanatory mechanism in the present study. Rather, the mediation analyses should be interpreted as theory-informed secondary analyses that tested a plausible pathway but yielded null findings. These results suggest that alternative mechanisms—such as affect regulation, self-regulatory conflict, stress, or sleep-related processes—may better explain the observed associations.

One possible explanation is that the context in which physical activity occurs may differ. Previous mediation studies have suggested that only certain domains of physical activity—such as leisure-time activity—are associated with mental health outcomes (Li et al., 2023). Recent reviews further emphasize that the direction and magnitude of the association between physical activity and mental health or mental disorders depend on the activity domain, and that promoting leisure-time physical activity may yield the greatest benefits for enhancing mental health and preventing mental disorders (Teychenne et al., 2025). In the present study, we assessed only total physical activity and did not distinguish between activity domains. A substantial proportion of students’ physical activity may have arisen from compulsory contexts, such as physical education classes, sport skill courses, or mandatory exercise tasks (e.g., required morning exercises or running check-ins). Such non-leisure activities may generate additional physical and psychological fatigue without providing sufficient stress-relief or restorative benefits.

### 4.3 Limitations

Several limitations should be acknowledged. First, the cross-sectional design precludes causal inference. Although we explored dose–response patterns using restricted cubic splines and examined potential mediation, the temporal ordering among exercise procrastination/exercise addiction, physical activity, and mental well-being cannot be established; reverse causation (e.g., poorer well-being influencing exercise-related behaviors) remains possible. Therefore, all findings in the present study should be interpreted as associative rather than causal, and the proposed pathways should be viewed as tentative and cross-sectional. Second, all variables were assessed via self-report questionnaires, which may introduce recall bias and social desirability bias, and may inflate or attenuate associations due to common-method variance. In particular, physical activity was measured using a brief self-report scale rather than device-based indicators, which may limit measurement precision. Third, the sample consisted of Chinese university students with a relatively narrow age range, which may restrict generalizability to other age groups, non-student populations, or different cultural contexts. Fourth, although we adjusted for relevant covariates, residual confounding cannot be ruled out. Unmeasured factors such as sleep quality, academic workload, mental health history, personality traits, or broader environmental constraints may partly explain the observed relationships. Fifth, the number of participants with very high exercise addiction scores may be limited in a general student sample, which could reduce power to detect non-linear effects or small indirect effects in mediation analyses. Finally, our findings are based on specific instruments (PES, EAI-R, PARS-3, WHO-5); results may vary with alternative operationalizations, cut-offs, or diagnostic criteria. Future studies should replicate these findings using longitudinal or experimental designs, multi-method assessment (e.g., accelerometry and ecological momentary assessment), and more diverse samples.

## 5 Conclusion

In this cross-sectional study of Chinese university students, exercise procrastination and exercise addiction showed distinct relationships with mental well-being. Exercise addiction was associated with lower mental well-being, whereas exercise procrastination displayed an inverted U-shaped association with mental well-being in spline analyses. Although exercise addiction and exercise procrastination were both related to physical activity in different patterns, the present findings did not support physical activity as an explanatory mediator of their associations with mental well-being in this sample. Taken together, these findings suggest that exercise procrastination and exercise addiction are not simply “more or less exercise,” but distinct behavior patterns that show different cross-sectional associations with mental well-being. Future research should clarify the temporal ordering and causal nature of these patterns using longitudinal or experimental designs and objective activity measures, and should explore alternative mechanisms (e.g., sleep, affect regulation, social comparison, or academic stress) that may link exercise-related behaviors to mental well-being.

## Data Availability

The raw data supporting the conclusions of this article will be made available by the authors, without undue reservation.
