# Supplementary material for: Associations of exercise procrastination and exercise addiction with mental well-being
Source: Front Psychol. 2026 May 8;17:1818526. doi: 10.3389/fpsyg.2026.1818526 (PMC13200607; doi:10.3389/fpsyg.2026.1818526)
Supplement: Supplementary file 1 [file Supplementary_file_1.docx]

**Supplemental Materials**

**Table S1. Outcomes by major**

| **Major** | **n** | **EAI** | **PIE** | **PA** | **WHO-5** |
| --- | --- | --- | --- | --- | --- |
|  |  | **Mean (SD)** | **Mean (SD)** | **Mean (SD)** | **Mean (SD)** |
| Natural sciences | 280 | 16.15 (5.54) | 16.78 (5.71) | 18.84 (19.89) | 13.39 (4.90) |
| Social sciences | 290 | 17.31 (6.05) | 17.18 (6.25) | 27.20 (24.77) | 13.54 (4.91) |
| Comparison (*p*) | - | 0.025 | 0.578 | <0.001 | 0.766 |

**Table S2. Outcomes by sex**

| **Sex** | **n** | **EAI** | **PIE** | **PA** | **WHO-5** |
| --- | --- | --- | --- | --- | --- |
|  |  | **Mean (SD)** | **Mean (SD)** | **Mean (SD)** | **Mean (SD)** |
| Male | 298 | 18.06 (5.84) | 16.43 (6.26) | 28.61 (24.88) | 13.22 (5.09) |
| Female | 272 | 15.30 (5.47) | 17.58 (5.62) | 17.04 (18.70) | 13.73 (4.68) |
| Comparison (*p*) | - | <0.001 | 0.010 | <0.001 | 0.074 |

**Table S3. Outcomes by grade**

| **Grade** | **n** | **EAI** | **PIE** | **PA** | **WHO-5** |
| --- | --- | --- | --- | --- | --- |
|  |  | **Mean (SD)** | **Mean (SD)** | **Mean (SD)** | **Mean (SD)** |
| Year 1 | 332 | 16.30 (5.90) a | 16.24 (5.97) a | 19.56 (20.65) a | 13.25 (4.94) a |
| Year 2 | 130 | 17.22 (6.03) a | 18.47 (5.97) b | 26.62 (23.89) b | 14.06 (5.18) a |
| Year 3 | 87 | 17.56 (5.54) a | 17.95 (5.76) b | 29.84 (26.39) b | 13.55 (4.51) a |
| Year 4 | 21 | 17.33 (3.98) a | 15.43 (5.18) ab | 29.14 (24.94) ab | 12.86 (3.77) a |
| Comparison (*p*) | - | 0.083 | <0.001 | 0.001 | 0.358 |

*Note:* different letters indicate statistically significance between groups; the between-group comparison has been adjusted using Bonferroni correction.
